# Camel-Associated Antimicrobial Resistance: An Overlooked One Health Interface

**DOI:** 10.3390/vetsci13040383

**Published:** 2026-04-15

**Authors:** Arwa A. Faizo, Thamir A. Alandijany

**Affiliations:** 1 Special Infectious Agents Unit, King Fahd Medical Research Center, King Abdulaziz University, P.O. Box 128442, Jeddah 21362, Saudi Arabia; aafaizo@kau.edu.sa; 2 Department of Medical Laboratory Sciences, Faculty of Applied Medical Sciences, King Abdulaziz University, P.O. Box 80324, Jeddah 21589, Saudi Arabia

**Keywords:** camel, antimicrobial resistance, one Health, zoonotic transmission, extended-spectrum beta-lactamase, colistin resistance

## Abstract

Camel-associated antimicrobial resistance (AMR) is an underrecognized component of the One Health landscape, particularly in the Gulf Cooperation Council (GCC) and North Africa. Available evidence demonstrates the presence of clinically significant resistance mechanisms in camel populations, including extended-spectrum beta-lactamases, carbapenemases, colistin resistance genes, and multidrug-resistant clones. Molecular similarities between camel and human isolates suggest potential cross-species transmission and highlight camels as possible reservoirs within interconnected human–animal–environment systems. Despite documented resistance patterns, camel production systems remain largely excluded from national AMR surveillance and stewardship frameworks. This gap reflects limited camel-specific data on antimicrobial usage and structural challenges related to pastoral mobility, cross-border trade, and emerging commercial intensification. Strengthening diagnostic capacity, implementing tailored antimicrobial stewardship strategies, and integrating camels into national AMR action plans are essential to mitigate potential public health risks. Proactive inclusion of camel systems within regional AMR governance frameworks is necessary to prevent further amplification of resistance.

## Simple Summary

Camels are an important source of food and livelihood in many regions, especially in the Middle East and North Africa. However, they may also carry bacteria that are resistant to antibiotics, which can pose risks to both animal and human health. This review highlights current evidence showing that antimicrobial resistance is present in camel populations, including resistance to critical antibiotics. Although the available data are still limited, some findings suggest possible transmission between camels, humans, and the environment. Despite these risks, camels are rarely included in national antimicrobial resistance monitoring and control programs. This article emphasizes the need to include camel production systems in One Health strategies, improve surveillance, and develop targeted antimicrobial stewardship approaches to reduce potential public health threats.

## 1. Introduction

Camels occupy a unique ecological, cultural, and economic niche across the Middle East, North Africa, and parts of Asia [1,2]. Despite close human interaction through meat production, raw milk consumption, live-animal markets, and slaughterhouse activities, their role in the emergence and dissemination of antimicrobial resistance (AMR) remains poorly characterized [3,4,5,6]. Within the One Health framework, camels may represent an underrecognized reservoir of antimicrobial-resistant pathogens and a potential interface for cross-species exposure with implications for food safety, occupational exposure, and possible cross-species transmission [3,4].

Unlike cattle, poultry, and swine—species central to global AMR surveillance—camels are typically raised in extensive pastoral systems characterized by herd mobility, cross-border trade, and limited veterinary oversight [1,2]. Simultaneously, the rapid expansion of commercial camel dairies and meat production has introduced new antimicrobial exposure dynamics without parallel development of species-specific stewardship frameworks [3,4,5,6,7]. These structural and ecological features distinguish camel husbandry from conventional livestock systems and may influence AMR dynamics in ways not fully captured by existing surveillance frameworks.

Available evidence, although limited, demonstrates the presence of clinically relevant resistant bacteria in camel-derived products [4,7,8,9,10,11,12,13]. However, most studies remain descriptive and geographically restricted, with limited data on transmission dynamics, molecular linkages between camel and human isolates, and resistance trends. Despite the growing economic and cultural importance of camel husbandry—particularly in regions where raw milk consumption is common—camels remain largely absent from national AMR surveillance and policy frameworks [1,7,9,11,14].

This review therefore aims not only to synthesize current evidence on antimicrobial resistance in camels but also to critically highlight the structural gaps in surveillance, policy integration, and stewardship design that limit effective intervention. By situating camel-associated AMR within a systems-level One Health framework, we highlight both the documented microbiological risks and the institutional factors that have contributed to their under-recognition.

## 2. Literature Search, Study Selection, and Data Extraction

The review is conducted as a narrative synthesis supported by a structured literature retrieval approach. It synthesizes published evidence on antimicrobial resistance (AMR) in camel populations and camel-associated bacteria, with particular focus on geographic distribution, resistance profiles, and ecological drivers within desert and semi-arid production systems. A structured literature retrieval approach was used to identify relevant studies, followed by predefined eligibility screening and standardized data extraction.

### 2.1. Literature Search Strategy

A structured literature search was conducted in February and March 2026 to identify studies reporting antimicrobial resistance (AMR) in camel populations and camel-associated bacterial isolates. Literature retrieval was performed using the Elicit research platform, which indexes and semantically searches records from Semantic Scholar and OpenAlex, covering more than 138 million academic publications. Two targeted semantic queries were used to capture complementary aspects of camel-associated AMR research. The first query focused on the prevalence and geographic distribution of antimicrobial resistance in camel populations across the Gulf Cooperation Council (GCC) and North African regions, while the second query targeted studies examining antimicrobial resistance profiles and ecological transmission dynamics of camel-associated bacteria in desert and semi-arid ecosystems. The search returned 50 records for each query, ranked by semantic relevance. This approach was intended to capture studies reporting antimicrobial resistance prevalence, bacterial resistance profiles, ecological drivers of resistance, and potential transmission pathways involving camel populations.

### 2.2. Eligibility Criteria

Retrieved records were screened according to predefined criteria addressing geographic relevance, target animal population, methodological rigor, and availability of microbiological resistance data. Studies were considered eligible if they met the following criteria:

1. Geographic Region: Studies conducted in GCC countries and/or North African countries?
2. Camel population: Studies involving camels (*Camelus dromedarius* or *Camelus bactrianus*) as the primary animal population.
3. Antimicrobial resistance focus: Studies investigating antimicrobial-resistant bacteria isolated from camel populations or camel-associated environments.
4. Microbiological data: Studies reporting antimicrobial susceptibility testing results using recognized laboratory methods such as disk diffusion, broth microdilution, or automated susceptibility systems.
5. Study design: Observational studies (cross-sectional, cohort, or case–control), experimental studies, surveillance reports, or reviews reporting relevant resistance data.
6. Quantitative resistance data: Studies providing measurable resistance prevalence, susceptibility results, or molecular resistance markers.
7. Sample size: Studies involving at least 10 animals when reporting case series or observational data, to reduce inclusion of highly anecdotal reports while retaining relevant camel-focused studies from a limited evidence base.

For analyses focusing on regional distribution, studies conducted in GCC countries (Bahrain, Kuwait, Oman, Qatar, Saudi Arabia, and the United Arab Emirates) or North African countries (Algeria, Egypt, Libya, Morocco, Sudan, and Tunisia) were prioritized. For analyses examining ecological and transmission dynamics, studies conducted in desert, semi-arid, or arid ecosystems were considered relevant.

Studies were excluded if they lacked antimicrobial susceptibility testing data, focused exclusively on antimicrobial usage without resistance testing, investigated non-bacterial resistance, or did not involve camel populations.

### 2.3. Study Selection

Titles and abstracts of the retrieved records were screened to determine relevance to the objectives of the review. Screening considered the eligibility criteria collectively, with emphasis on the presence of antimicrobial resistance testing data in camel populations.

Studies addressing either resistance prevalence in camel populations or microbiological and ecological characteristics of camel-associated antimicrobial-resistant bacteria were included. When abstracts did not provide sufficient information for eligibility assessment, the full text was reviewed. Given the narrative nature of this review, formal risk-of-bias assessment and duplicate removal procedures typical of systematic reviews were not applied.

### 2.4. Data Extraction

Data were extracted mainly from 20 records using a structured framework to ensure consistent capture of study characteristics, antimicrobial resistance findings, and ecological factors associated with camel production systems, with additional relevant studies identified through reference screening (Table 1).

**Table 1:** Below displays the extracted variables.

Category	Extracted Variables
Study characteristics	Geographic location (country, region), ecosystem context (desert, semi-arid, arid), study setting (farms, abattoirs, retail, environmental sampling), sampling period, study design, and sample size
Camel population characteristics	Camel species (dromedary or Bactrian), population characteristics (age, sex, health status when available), herd management systems, antimicrobial exposure history, and contact with livestock, wildlife, or humans
Sample sources	Biological sample types (milk, feces, meat, nasal swabs, blood, urine, skin), environmental samples (water, soil, feed, facilities), and sampling approaches
Antimicrobial resistance prevalence	Overall AMR prevalence, prevalence by bacterial species and sample type, multidrug resistance prevalence, and detection of extended-spectrum β-lactamase (ESBL) producers
Bacterial organisms	Bacterial genera and species identified, distribution of isolates, and detection of clinically relevant pathogens such as methicillin-resistant *Staphylococcus aureus*
Resistance patterns and mechanisms	Antibiotics tested and resistance rates, antibiotic classes with the highest resistance prevalence, resistance genes and mobile genetic elements, and multidrug resistance profiles
Transmission and ecological factors	Evidence of transmission pathways (animal-animal, animal–human, environmental), environmental persistence, seasonal patterns, and horizontal gene transfer
Comparative findings	Comparisons between camels and other livestock species regarding resistance prevalence, bacterial distribution, and ecological roles
Methodological characteristics	Detection methods (culture or molecular), antimicrobial susceptibility testing techniques, interpretation criteria, sampling strategies, and reported methodological limitations

### 2.5. Evidence Synthesis

Extracted data were summarized descriptively to identify patterns in antimicrobial resistance prevalence, bacterial species distribution, resistance determinants, and ecological drivers associated with camel production systems. Particular attention was given to the geographic distribution of resistance, resistance profiles of camel-associated bacteria, and environmental or management factors influencing antimicrobial resistance dynamics in desert and semi-arid ecosystems. Findings were interpreted according to data type (prevalence, phenotypic resistance, genotypic detection, or food-interface contamination) to avoid inappropriate cross-study comparisons.

## 3. Prevalence and Geographic Distribution of AMR in Camels

Assessing the prevalence and geographic distribution of antimicrobial resistance in camel populations is essential for determining whether camels act as localized hosts or broader regional reservoirs of resistant bacteria. Although available prevalence estimates provide an initial indication of the scale of the issue, comparisons across studies remain challenging and should be interpreted cautiously due to differences in sampling strategies, study design, and laboratory methodologies. Some current evidence from the GCC and North Africa is summarized in Table 2. The evidence presented in this section includes heterogeneous data types, including animal-level prevalence, isolate-level resistance frequencies, genotypic detection, and food-interface contamination data, which are not directly comparable and should be interpreted within their respective methodological contexts.

Antimicrobial resistance is reported across multiple studies in camel populations across GCC and North African countries, with resistant bacteria detected in 22–70% of camels depending on the microorganism and geographic location [10,11,18]. These estimates should be interpreted cautiously due to differences in study design, sampling sources, and laboratory methodologies. Extended-spectrum beta-lactamase producers ranged from 1.3% to 45% of isolates [11,12,18], while multidrug resistance affected 22–50% of bacterial strains [7,15,16], which represents isolate-level resistance data and should be interpreted within the context of study-specific sampling and laboratory methodologies. The mcr-1 gene conferring colistin resistance was identified in camels from the United Arab Emirates and Tunisia [10,18], representing the first documentation of this resistance mechanism in Gulf region camels. Resistance was highest against penicillins and tetracyclines across both regions [7,15,16,19].

The limited comparative data suggest resistance patterns in camels, which are similar to, but not dramatically worse than, those of other livestock. The only head-to-head comparison found camels carrying *S. aureus* more often than cattle or horses (53% vs. 15%) [20], but less often than sheep carrying methicillin-resistant strains (4.4% vs. 9.3%) [20]. The key uncertainty is whether camels truly represent a greater resistance reservoir than other livestock, or if they have simply been studied less intensively. Most studies only examined camels without comparing them to cattle, sheep, or goats raised in the same conditions, making it impossible to know if high resistance rates are camel-specific or reflect broader agricultural practices. Sample sizes were often small (as few as 46 animals) [17,21], and many studies used convenience sampling from single farms or slaughterhouses rather than random selection across regions [10,11,12]. The open question is whether interventions should specifically target camels or instead address antibiotic use across all livestock in these regions.

## 4. Antimicrobial Resistance Profiles in Camel-Associated Bacteria

While prevalence estimates provide insight into the magnitude of antimicrobial resistance, characterization of specific resistance phenotypes and genotypes is critical for assessing clinical relevance and zoonotic potential. The types of resistance mechanisms identified—particularly those affecting critically important antimicrobials such as third-generation cephalosporins, carbapenems, and colistin—determine whether camel-associated bacteria represent localized agricultural concerns or broader public health threats.

AMR prevalence in camels varies substantially across transmission interfaces, though evidence remains limited to meat processing and raw milk consumption pathways [3,4,7]. Raw milk consumption showed the highest resistance rates, with 80% of bacterial isolates resistant to multiple antibiotic classes including penicillin, tetracyclines, and carbapenems in Kuwait [4,7]. Multidrug-resistant pathogens including *Klebsiella pneumoniae*, *Escherichia coli*, and *Enterobacter hormaechei* were identified in raw milk samples [7], with antibiotic resistance genes mediating resistance to 18 different antibiotic classes [7]. In addition to consumption practices, camel milk composition—including its high fat content, presence of antimicrobial proteins such as lactoferrin and lysozyme, and unique physicochemical properties—may influence bacterial survival, persistence, and selective pressures [14], although direct evidence linking these factors to antimicrobial resistance dynamics remains limited. Camel milk has been shown to harbor antimicrobial resistance genes (ARGs) and multidrug-resistant bacterial populations. For example, the presence of ARGs conferring resistance to multiple antibiotic classes, including fluoroquinolones, tetracyclines, macrolides, and glycopeptides, was reported with many genes associated with mobile genetic elements such as plasmids and transposons [7]. These findings suggest a potential for horizontal gene transfer following ingestion. Indeed, horizontal gene transfer is a well-established mechanism for the dissemination of antimicrobial resistance within the human gut microbiome [22]. However, direct evidence linking camel milk consumption to resistance selection or gene transfer within the human gut microbiota remains lacking.

Meat processing interfaces showed lower prevalence rates of 8–20% for specific resistant pathogens, including MRSA in 20% of Saudi Arabian meat samples, VRSA in 14.5% and *Pseudomonas spp.* in 10% of Egyptian samples, and ESBL-producing *E. coli* in 8–11.3% of samples [3,23,24,25,26,27]. Similar findings have been reported in other regions, including *Acinetobacter baumannii* and *Staphylococcus aureus* in Iran, and a high prevalence of *Vibrio parahaemolyticus* (~33%) in Libya [28,29,30], collectively highlighting the food-chain transmission potential of resistant and pathogenic bacteria associated with camel meat.

These differences likely reflect methodological variation—comprehensive bacterial community assessment in milk versus targeted pathogen surveillance in meat—rather than solely biological differences in resistance burden. Direct contact with live camels and water contamination interfaces lacks quantitative prevalence data, representing critical surveillance gaps. There is a lack of implemented integrated One Health surveillance connecting AMR patterns across camel, human, and environmental domains, preventing assessment of cross-interface transmission dynamics and limiting conclusions about interconnected AMR risks across the camel–human–environment interface (Table 3).

**Table 3:** Demonstrates studies that examined antimicrobial-resistant bacteria in camels across desert and semi-arid ecosystems. Studies were conducted between 2015 and 2024 across North Africa, East Africa, and the Middle East. Studies primarily focused on dromedary camels, with most of the sampling being fecal material, though nasal swabs, milk, meat, and environmental samples were also examined.

Geographic Location	Ecosystem Type	Sample Size	Primary Bacteria Studied	Sample Types	Management System	Ref.
Northern Kenya	Arid and semi-arid lands	304 camels	*Escherichia coli*	Fecal	Extensive (pastoral) and intensive (ranch)	[31]
Somalia and Kenya	Arid and semiarid regions	84 camels, 7 cattle	Staphylococcaceae	Not specified	Not mentioned	[32]
Northern Kenya	Arid and semi-arid lands	123 *E. coli* isolates	*Escherichia coli*	Fecal	Pastoral	[33]
Egypt	Semi-arid/arid	121 fecal swabs	*Escherichia coli*	Fecal	Intensive/mixed (abattoir samples)	[8]
Tunisia	Arid regions	232 camels	*E. coli*, *K. pneumoniae*	Fecal	Tourist and meat-producing sectors	[18]
Egypt	Desert/semi-arid	200 camels	*Pseudomonas aeruginosa*	Meat	Intensive/mixed (abattoir samples)	[12]
Saudi Arabia	Desert/semi-arid	100 camel sample	*Escherichia coli*	Cecal contents	Not mentioned	[11]
Kuwait	Desert	8 pooled milk samples	Multiple species	Milk	Not specified	[7]
Egypt	Semi-arid/arid	40 camels	MRSA	Nasal, milk, soil, water	Not specified	[34]
Algeria	Semi-arid and arid	46 camels	MRS/MRM	Nasal	Pastoral setting	[17]

### 4.1. Escherichia coli Resistance Patterns

*E. coli* was the most frequently studied bacterial species. Detection rates in fecal samples ranged from 26.0% in Saudi Arabia [11] to 70.3% in Tunisia [18], with Northern Kenya reporting 40.46% [33]. Beta-lactam resistance was consistently high across studies. In Northern Kenya, resistance to cefaclor reached 28.5%, cefotaxime 16.3%, and ampicillin 9.7% [31]. A parallel study in the same region reported similar patterns: cefaclor 28.46%, cefotaxime 16.26%, ampicillin 9.76%, and ceftazidime 8.13%. Additional resistance to tetracycline was observed at 4.87% [33].

ESBL-producing *E. coli* prevalence varied substantially. In Northern Kenya, ESBL producers comprised 3.3% of isolates [31], while Saudi Arabia reported 26.9% ESBL prevalence in camel samples [11]. The dominant ESBL genes identified were blaCTX-M-15 and blaCTX-M-27 [18,31,33]. Multiple blaTEM variants were also detected, including blaTEM-1, blaTEM-11, blaTEM-104, blaTEM-214, and blaTEM-243 [18,31,33]. ESBL-producing *E. coli* were associated with phylogenetic groups B1, B2, and D [31], indicating diverse genetic backgrounds. The detection of the *E. coli* O25b:H4-ST131 clone in both camel and human isolates in Saudi Arabia represents a concerning finding given its well-established association with multidrug-resistant human infections [11]. Indeed, ST131 is a globally disseminated multidrug-resistant lineage strongly associated with human extraintestinal infections, particularly urinary tract infection and invasive disease [35]. Although this finding does not by itself prove direct camel-to-human transmission, it supports the possibility of shared AMR ecology or epidemiological linkage across camel and human interfaces.

### 4.2. Carbapenem and Colistin Resistance

Egypt reported particularly concerning resistance patterns. Among 75 *E. coli* isolates from camels, 27 were carbapenemase-producing, representing 36% prevalence [8]. Carbapenemase genes included blaOXA-48 (*n* = 7), blaNDM (*n* = 14), and blaVIM (*n* = 6) [8]. Colistin resistance genes were detected in 29 isolates, with mcr-3 being most prevalent (*n* = 21), followed by mcr-4 (*n* = 3), mcr-1 (*n* = 3), and mcr-2 (*n* = 2) [8]. The first description of the mcr-1 gene in a meat-producing camel was reported from Tunisia, highlighting the potential involvement of camels in the dissemination of emerging resistance mechanisms [18].

### 4.3. Staphylococcaceae Resistance

Methicillin-resistant Staphylococci (MRS) and Mammaliicoccus (MRM) were identified in 57% of Algerian farms sampled [17]. The predominant species were *M. lentus*, *S. epidermidis*, and *S. aureus* [17]. Three MRSA isolates belonged to ST6, spa type t304, while MRSE ST61 was the predominant sequence type [17]. A novel finding was the detection of an SCCmec-mecC hybrid element in *M. lentus*, representing the first such report in this species [17]. Resistance genes identified included mecA, mecC, ermB, tet(K), and blaZ [17]. This finding is notable because hybrid resistance elements may facilitate the dissemination of methicillin resistance determinants across staphylococcal and related lineages, thereby increasing their clinical and ecological relevance beyond a single host species.

In Egypt, MRSA prevalence varied by sample type: 12.5% in nasal swabs, 6.67% in milk, 13.3% in soil, and 6.67% in water [12]. All MRSA isolates displayed 100% resistance to cefoxitin and penicillin, and 80% resistance to gentamicin [12]. East African Staphylococcaceae strains from camels and cattle showed resistance to tetracycline, benzylpenicillin, oxacillin, erythromycin, clindamycin, trimethoprim, gentamicin, and streptomycin, with about one-third displaying non-wild-type MICs [32]. The first methicillin- and multidrug-resistant camel *S. epidermidis* strain of sequence type ST1136 was identified in East Africa [32].

### 4.4. Pseudomonas aeruginosa Resistance

In Egyptian camel meat, *P. aeruginosa* was isolated at 22.5% prevalence, with 45% of isolates being ESBL-producers [12]. These isolates showed high-level resistance to ceftazidime, ceftriaxone, and rifampicin, with lower resistance to meropenem, amikacin, imipenem, gentamicin, and ciprofloxacin [12]. ESBL genotypes included blaPER-1, blaCTX-M, blaSHV, and blaTEM [12].

### 4.5. Multidrug Resistance

The multidrug resistance (MDR) burden varied significantly across sample types and countries. In Kuwait, 33% of bacterial isolates from raw camel milk were classified as MDR, defined as resistance to at least three antibiotic classes [4]. Shotgun sequencing of Kuwaiti camel milk revealed resistance genes to 18 antibiotic classes, with the highest resistance to fluoroquinolones (12.48%) and disinfecting agents and antiseptics (9%) [7]. Phenotypic testing showed 80% of isolates were resistant to multiple antibiotic classes, with the highest resistance to penicillin, tetracyclines, and carbapenems [7]. Multidrug-resistant pathogens including *K. pneumoniae*, *E. coli*, and *Enterobacter hormaechei* comprised 33% of isolates [7].

In Saudi Arabia, a study reported an MDR index of 0.13 for camel isolates compared to 0.17 for human isolates, suggesting a moderate but measurable MDR burden in camels that approaches levels seen in human populations [11]. Whole-genome sequencing of MAP isolates from Saudi Arabian camels identified 34 distinct AMR genes, along with 10 virulence genes, demonstrating the complex genetic architecture underlying antimicrobial resistance in camel-associated pathogens [11].

## 5. Transmission Dynamics and Ecological Significance

The presence of resistant bacteria in camel populations alone does not confirm their epidemiological significance. The critical question is whether these organisms and resistance determinants move across species boundaries and environmental compartments, and current evidence remains suggestive rather than definitive (Table 4). Evaluating transmission dynamics is necessary to determine whether camels function as passive carriers or active amplifiers within regional AMR networks.

**Table 4:** Evidence of zoonotic transmission risk.

Evidence Type	Pathogen/Resistance Mechanism	Location	Findings	Reference
Molecular typing	*E. coli* ST131 clone	Saudi Arabia	Identical drug-resistant clone detected in two camel and two human isolates	[11]
ESBL detection	ESBL-producing *E. coli*	Saudi Arabia	ESBLs present in both camel (26.9%) and human (36.4%) samples	[11]
Phylogenetic analysis	MAP strains	Saudi Arabia	Clade 3 includes human-associated strains, suggesting zoonotic implications	[9]
Contaminated products	Antibiotic Resistance Genes (ARG) in camel milk	Kuwait	Presence of ARGs including resistance to critically important antibiotics	[7]
Antibiotic residues	Residues in meat	Middle East (multiple countries)	Antibiotic residues detected, indicating potential for resistance selection	[3]

### 5.1. Evidence of Horizontal Gene Transfer

Multiple studies documented mobile genetic elements facilitating resistance gene dissemination [8,10,15,18,36]. Kuwaiti camel milk samples showed antibiotic resistance genes carried on plasmids surrounded by mobile genetic elements [7], indicating potential for horizontal transfer. In East Africa, potential horizontal gene transfers were identified between camel and cattle strains and across distinct Staphylococcaceae clades and species [32].

### 5.2. Clonal Relationships and Zoonotic Potential

Direct evidence of animal-to-human transmission potential emerged from Saudi Arabia, where the same *E. coli* O25b:H4-ST131 drug-resistant clone was detected in both camel cecal contents and human urinary tract infection samples [11]. This finding suggests camels serve as reservoirs for human pathogenic strains. The presence of MRSA ST6/t304 in Algerian camels, a strain associated with human infections across multiple regions, further supports zoonotic transmission pathways [17]. Similarly, a study characterized the nasal Staphylococcaceae microbiota of dromedary camels in Algeria and identified MRSE ST61, which has documented associations with human disease [17].

### 5.3. Environmental Persistence

Environmental sampling revealed MRSA in soil (13.3% detection) and water (6.67% detection) surrounding camels, demonstrating environmental contamination and potential persistence [34]. This environmental reservoir may facilitate transmission between animals and humans beyond direct contact [34]. Furthermore, the detection of resistance genes in camel milk creates a direct pathway for human exposure through consumption, particularly of raw milk [7]. The presence of multidrug-resistant *K. pneumoniae* and *E. coli* in milk samples poses immediate public health risks [7].

## 6. Drivers of Antimicrobial Exposure in Camel Production Systems

Studies of general livestock production across Kenya, Uganda, India, and Sudan identified consistent drivers that may partially apply to camel systems [37,38,39,40,41]. Economic pressures including high demand for animal products and commercialization drove antimicrobial use, while disease management needs—particularly mastitis and other production-limiting infections—created therapeutic demand [37,38,39,40,41]. Informal distribution systems dominated in many settings, with antimicrobials frequently sold without prescriptions and over-the-counter access common [37,38,39,40,41]. Infrastructure limitations, including scarce and expensive professional veterinary services, led farmers to rely on self-treatment or untrained providers [37,38,39,40,41]. Knowledge gaps regarding antimicrobial resistance were also documented in several regions [37,38,39,40,41]. Regarding prophylactic versus therapeutic use, available evidence from broader livestock systems suggests therapeutic use predominates, although prophylactic practices may occur in high-disease-pressure settings with limited veterinary oversight [37,39,40].

Whether similar patterns exist in camel production remains unknown due to the absence of camel-specific antimicrobial usage data, representing a critical surveillance gap. Emerging evidence from camel-specific settings provides preliminary insights into antimicrobial usage practices. A cross-sectional study conducted among camel dairy farmers in the Banadir region of Somalia reported widespread misuse of antibiotics, including use without veterinary prescription (80%) and administration without veterinary supervision (88%) [42]. The study also highlighted substantial knowledge gaps, with 70% of farmers lacking awareness of antimicrobial resistance, alongside practices such as consumption of milk during antibiotic treatment without adherence to withdrawal periods [42]. However, these findings are based on a single geographically restricted study and may not be generalizable across different camel production systems. The absence of large-scale, multi-regional, or longitudinal studies on antimicrobial usage in camels remains a major limitation, restricting the ability to accurately assess drivers of resistance at the population level. Indeed, the lack of systematic data on prescribing practices, drug classes used, treatment duration, and residue monitoring in camel systems prevents accurate assessment of selection pressures driving observed resistance patterns [43]. Addressing camel-associated AMR therefore requires not only microbiological surveillance but also targeted investigation of antimicrobial usage practices specific to camel husbandry contexts.

This is particularly important as antibiotic residue risks represent an underexplored dimension of camel-associated AMR. Residues of commonly used antimicrobials such as penicillin and oxytetracycline have been reported to persist in camel milk at concentrations exceeding maximum residue limits (MRLs) for extended periods, potentially creating selective pressure for resistant bacteria [44]. For example, penicillin residues have been detected at levels up to 7.5-fold above established MRLs several weeks after administration, while oxytetracycline residues may persist above acceptable limits for up to two weeks [44].

From a mechanistic perspective, even sub-inhibitory antibiotic concentrations can exceed minimal selective concentrations (MSC), favoring the enrichment of resistant bacterial populations [45]. Experimental studies have demonstrated that low-level antibiotic residues in milk can select for resistant *Escherichia coli* in vivo [46], although direct evidence linking camel milk residues to resistance selection in the human gut microbiota remains limited.

Collectively, these findings highlight antibiotic residue monitoring as an important but underdeveloped component of AMR surveillance and stewardship in camel production systems, particularly in settings where raw milk consumption and informal distribution pathways are common.

## 7. Toward Surveillance and Stewardship of Camel-Associated AMR

Translating microbiological evidence into effective AMR control measures requires functional surveillance systems and context-appropriate stewardship strategies. However, surveillance models developed for sedentary intensive livestock systems may not be directly applicable to mobile pastoral camel herds in arid and semi-arid regions [38,41]. Pastoral mobility presents a major structural challenge, as frequent herd movement across large geographic areas and national borders complicates herd identification, repeated sampling, traceability, and continuity of surveillance efforts. It also limits consistent veterinary oversight, follow-up of treated animals, and implementation of standardized antimicrobial stewardship interventions across different administrative regions. Consequently, the feasibility and scalability of AMR monitoring and intervention strategies in camel production systems remain largely untested from national AMR surveillance and stewardship frameworks [3].

Current evidence for AMR surveillance in camels is extremely limited and provides little insight into pastoral herd management contexts, sampling strategies, or integration with veterinary practices [3,9,13]. Although antimicrobial stewardship and regulation of veterinary antibiotic use have been recommended, no camel-specific interventions have been implemented or evaluated [38,47]. As a result, several operational challenges remain unresolved, including how surveillance can be conducted in mobile herds, how veterinary access and drug quality can be ensured in remote regions, and how pastoral communities can be effectively engaged in AMR mitigation. Feasible approaches may include phased baseline surveillance to establish initial resistance profiles, followed by sentinel-site monitoring in representative camel production areas. Integration of sampling into existing infrastructure—such as slaughterhouses, live-animal markets, and milk collection points—may improve practicality and coverage. Targeted sampling strategies, including periodic milk and fecal surveillance, could provide cost-effective insights into resistance patterns. In addition, linkage with mobile veterinary outreach services may facilitate sample collection, data reporting, and implementation of context-appropriate stewardship interventions in pastoral settings.

The absence of tested surveillance and control strategies therefore represents not only a research gap but also a potential governance vulnerability within regional One Health frameworks. This gap likely reflects several structural factors. Existing stewardship guidelines are primarily designed for conventional livestock and provide limited operational guidance for camel husbandry, which often involves extensive grazing systems, harsh environmental conditions, and culturally embedded practices such as raw milk consumption [3,6,48]. Limited diagnostic infrastructure and surveillance capacity further constrain implementation. Addressing camel-associated AMR will therefore require stewardship frameworks specifically adapted to camel production systems. Preventive measures—including improved veterinary access, vaccination programs, hygiene standards in milk and meat production, and targeted education for pastoral communities—may represent more feasible interventions than intensive monitoring alone [3,6,48].

Implementation should follow a phased approach beginning with strengthened diagnostic capacity and baseline surveillance, followed by pilot stewardship programs across representative camel production systems and eventual development of harmonized regional guidelines supported by One Health coordination.

## 8. Conclusions

Evidence now indicates that camels harbor clinically significant antimicrobial resistance mechanisms, including multidrug-resistant strains and resistance genes of critical public health concern. Molecular similarities between camel and human isolates suggest that camel production systems may function as a potential active interface within regional AMR networks rather than isolated reservoirs.

Despite this, camels remain largely absent from national AMR surveillance and stewardship frameworks. This gap reflects a structural misalignment between conventional livestock-based policies and the ecological realities of camel husbandry, including pastoral mobility, emerging intensification, and culturally embedded consumption practices.

Beyond regional implications, camel-associated AMR also has relevance for global health security, as camel production systems are closely linked to cross-border trade, animal movement, and international travel, particularly in regions with high mobility and tourism activity. These interconnected pathways may facilitate the transboundary spread of resistant bacteria and resistance genes, underscoring the importance of integrating camel systems into broader One Health surveillance and control strategies. Without deliberate inclusion, camel-associated AMR risks may persist as a preventable but overlooked component of regional resistance ecology.

## Figures and Tables

**Table 2:** Some studies on antimicrobial resistance in camel populations across the Middle East and North Africa.

Study	Study Design	Findings
A. Fadlelmula et al., 2016 [11]	Compared *E. coli* from 100 camel intestines and 100 human urinary infections in Saudi Arabia	Found resistant *E. coli* in 26% of camels and 33% of humans; 27% of camel isolates produced ESBLs (enzymes that break down antibiotics)
M. M. Ali et al., 2024 [15]	Tested 200 milk samples from camels with mastitis (udder infection) in Egypt	Found *S. aureus* bacteria in 30% of samples; half were multidrug-resistant, and all carried the gene for methicillin resistance
Imane Barka et al., 2023 [16]	Examined 200 lactating camels in Algeria for mastitis	35% had mastitis; among bacteria isolated, 65% were resistant to at least one antibiotic and 22% were multidrug-resistant
Chahrazed Belhout et al., 2023 [17]	Collected nasal swabs from 46 camels on 7 farms in Algeria	Found methicillin-resistant bacteria in 57% of farms; discovered a novel genetic element that spreads resistance
M. Elhariri et al., 2017 [12]	Tested meat from 200 healthy camels at Egyptian slaughterhouses	Found Pseudomonas bacteria (which can cause serious infections) in 22.5% of samples; 45% of these produced ESBLs
A. Ghazawi et al., 2024 [10]	Analyzed feces from 50 camels on a Dubai farm	22% carried *E. coli* with the mcr-1 gene (makes bacteria resistant to colistin, a last-resort antibiotic); first report of this gene in Gulf camels
Mariem Saidani et al., 2019 [18]	Collected fecal samples from 232 camels in Tunisia over 2 years	Found *E. coli* in 70% of camels; very few (1.3%) produced ESBLs, but one meat-producing camel carried the mcr-1 gene
Mayada Gwida et al., 2019 [19]	Examined feces, meat, and liver from 100 camels at an Egyptian slaughterhouse	Found Campylobacter in 20–33% and *S. aureus* in 15–45%, depending on sample type; all were resistant to rifampicin but sensitive to enrofloxacin
Rita Rahmeh et al., 2024 [7]	Analyzed 8 pooled milk samples from retail stores in Kuwait	Found genes for resistance to 18 antibiotic classes; 80% of bacteria were resistant to multiple drugs, with the highest resistance to penicillin and carbapenems
A. Agabou et al., 2017 [20]	Swabbed noses of 45 camels, 43 sheep, 40 cattle, 33 horses, and 2 monkeys in Algeria	Camels had 53% carriage of *S. aureus* vs. 44% in sheep, 15% in horses and cattle; methicillin-resistant strains were found in 4.4% of camels and 9.3% of sheep

Note: Reported values represent study-specific measures (e.g., animal-level prevalence, isolate-level resistance, or gene detection) and are not directly comparable across studies due to methodological heterogeneity.

## Data Availability

No new data were created or analyzed in this study. Data sharing is not applicable to this article.

## Abbreviations

AMR	Antimicrobial resistance
ESBL	Extended-spectrum beta-lactamase
MDR	Multidrug resistance
MRSA	Methicillin-resistant Staphylococcus aureus
MRS	Methicillin-resistant Staphylococci
MRM	Methicillin-resistant Mammaliicocci
ARG	Antibiotic resistance gene
GCC	Gulf Cooperation Council
